# Randomised phase II trial of gemcitabine plus vinorelbine *vs* gemcitabine plus cisplatin *vs* gemcitabine plus capecitabine in patients with pretreated metastatic breast cancer

**DOI:** 10.1038/bjc.2011.86

**Published:** 2011-03-15

**Authors:** H J Stemmler, D diGioia, W Freier, H W Tessen, G Gitsch, W Jonat, W Brugger, E Kettner, W Abenhardt, H Tesch, H J Hurtz, S Rösel, O Brudler, V Heinemann

**Affiliations:** 1 Med. Department III, Ludwig-Maximilians University of Munich, Campus Grosshadern, Munich, Germany; 2 Oncological Practice, Hildesheim, Germany; 3 Oncological Practice, Goslar, Germany; 4 Department of Gynaecology & Obstetrics, University of Freiburg, Freiburg, Germany; 5 Department of Gynaecology & Obstetrics, University of Kiel, Kiel, Germany; 6 Med. Department II, Clinical Centre Villingen-Schwenningen, Villingen-Schwenningen, Germany; 7 Department of Hematology & Oncology, Clinical Centre Magdeburg, Magdeburg, Germany; 8 Oncological Practice, Munich, Germany; 9 Oncological Practice, Frankfurt, Germany; 10 Oncological Practice, Halle, Germany; 11 Oncological Practice, Gütersloh, Germany; 12 Oncological Practice, Augsburg, Germany

**Keywords:** gemcitabine, cisplatin, vinorelbine, capecitabine, anthracycline resistance, metastatic breast cancer

## Abstract

**Background:**

An increasing proportion of patients are exposed to anthracyclines and/or taxanes in the adjuvant or neoadjuvant setting. Re-exposure in the metastatic stage is limited by drug resistance, thus evaluation of non-cross-resistant regimens is mandatory.

**Methods:**

Anthracycline-pretreated patients were randomly assigned to three gemcitabine-based regimens. Chemotherapy consisted of gemcitabine 1.000 mg m^−2^ plus vinorelbin 25 mg m^−2^ on days 1+8 (GemVin), or plus cisplatin 30 mg m^−2^ on days 1+8 (GemCis), or plus capecitabine 650 mg m^−2^ b.i.d. orally days 1–14 (GemCap), q3w. The primary end point was response rate.

**Results:**

A total of 141 patients were recruited on the trial. The overall response rates were 39.0% (GemVin), 47.7% (GemCis) and 34.7% (GemCap). Median progression-free survival was estimated with 5.7, 6.9 and 8.3 months, respectively. Corresponding median survival times were 17.5 (GemVin), 13.0 (GemCis) and 19.4 months (GemCap). Neutropenia ⩾grade 3 occurred in 16.7% (Gem/Vin), 4.4% (GemCis) and 0% (Gem/Cap), whereas non-haematological toxicities were rarely severe except grade 3 hand–foot syndrome in 2.0% of the GemCap patients (per patient analysis).

**Conclusions:**

This randomised phase II trial has revealed comparable results for three gemcitabine-based regimens regarding treatment efficacy and toxicity. Gemcitabine-based chemotherapy appears to be a worthwhile treatment option for pretreated patients with metastatic breast cancer.

Strategies in patients with advanced breast cancer are confounded by the increasing exposure of patients to chemotherapy in the adjuvant setting. Nevertheless, two general strategies are apparent and should be followed: (1) improving treatment efficacy by exploring new drugs and drug combinations, and (2) ensuring that efficacy is improved with the lowest cost to quality of life.

Gemcitabine as a single agent has induced overall response rates of 0–37% in first-line treatment, whereas the response rates in the second- or third-line therapy were 26 and 13% (Brodowicz *et al*, 2000; Spielmann *et al*, 2001; Blackstein *et al*, 2002; Heinemann, 2003, 2005; Modi *et al*, 2005). In studies limited to second- or third-line therapy after anthracycline and/or taxane exposure, response rates of 0–29% and median time to progression of 2–6 months were achieved (Spielmann *et al*, 2001; Modi *et al*, 2005; Heinemann *et al*, 2006; Seo *et al*, 2007). Several considerations support the use of gemcitabine and a platinum salt in the salvage treatment of metastatic breast cancer (MBC): First, *in vitro* studies indicate additive or synergistic activity that was most pronounced in platinum-resistant cell lines and was found to be due to an increased formation and an impaired repair of platinum–DNA adducts (Peters *et al*, 1995; van Moorsel *et al*, 1997). Second, gemcitabine and the platinum salts are usually not included into adjuvant or neoadjuvant chemotherapy. Therefore, resistance to either drug is unlikely to occur. Third, studies investigating the combination have shown minimal overlapping toxicity, suggesting an acceptable toxicity profile even in intensively pretreated patients. The combination of gemcitabine and cisplatin was shown to be effective in several trials, inducing response rates between 30 and 52% in patients pretreated with taxanes and/or anthracyclines (Kolaric and Vukas, 1991; Chitapanarux *et al*, 2006; Fuentes *et al*, 2006; Heinemann *et al*, 2006; Kim *et al*, 2008).

Vinorelbine has shown good efficacy as first-line treatment (40–60%), activity after anthracycline pretreatment has only been moderate (16%) (Degardin *et al*, 1994; Jones *et al*, 1995; Gregory and Smith, 2000). The dose-limiting toxicity of vinorelbine is in the form of non-cumulative haematotoxicity. The potential of adding gemcitabine to vinorelbine has been explored in various phase II studies (Nicolaides *et al*, 2000; Valenza *et al*, 2000; Park *et al*, 2009; Shehata *et al*, 2010). Haider *et al* (1999) conducted a study that evaluated gemcitabine plus vinorelbine (GemVin) separately as first-line therapy. In the subgroup of 45 chemonaive patients the overall response rate was 56%. Two additional studies that applied GemVin for second-line therapy (after anthracyclines±taxanes) achieved response rates of 48 and 54%, respectively (Haider *et al*, 1999; Valenza *et al*, 2000; Stathopoulos *et al*, 2002; Shehata *et al*, 2010). These studies demonstrate that the combination of GemVin is active not only as first-line treatment but also after pretreatment with anthracycline- or anthracycline/taxane-based regimens. Gemcitabine and capecitabine (GemCap) are among the few agents active in patients with MBC progressing after therapy with anthracyclines and taxanes. Andres *et al* (2005) conducted a phase II trial of GemCap in patients with disease progression after treatment with anthracyclines and taxanes. The response rate was 48.7%. Median time to progression was 5 months (range, 1–26 months) and the median overall survival duration was 10 months. Another study by Ciruelos *et al* (2009) investigated the combination in the first- and second-line setting with a response rate of 61 and 48.5%, respectively. Most frequent grade 3–4 observed toxic effects were neutropenia (60%) and hand–foot syndrome (16%). The authors concluded that the combination of GemCap is an active and safe regimen in anthracycline-pretreated breast cancer patients (Campos *et al*, 2001; Schilsky *et al*, 2001; Andres *et al*, 2005; Ciruelos *et al*, 2009; Malmstrom *et al*, 2010).

Taken together, these data provided the basis for this open, randomised phase II study, which investigated the efficacy of three different gemcitabine-based regimens for patients with MBC.

## Patients and methods

### Patient population

One-hundred and forty-one patients with histologically confirmed MBC were recruited on a treatment protocol approved by the independent ethics committees of all participating centres and was conducted according to the International Conference on Harmonisation (ICH) – Good Clinical Practice (GCP) guidelines. All patients were required to give written informed consent before study entry.

The study was registered by the US National Institutes of Health (http://clinicaltrials.gov/ ClinicalTrials.gov Identifier: NCT00480597).

Patients eligible for the trial required one previous anthracycline-based regimen (in the adjuvant or in the metastatic setting). There was no limit on number of previous chemotherapy regimens (except gemcitabine-, vinorelbine-, cisplatin- or capecitabine-containing regimens), or on the number of previous hormonal therapies. Moreover, immunotherapy or local radiotherapy was allowed. Patients were required to have at least one bi-dimensionally measurable lesion outside a previous radiation port. Other eligibility criteria included age ⩾18 and ⩽70 years, Karnofsky performance status ⩾70%, minimal life expectancy of 12 weeks, and adequate haematological, renal, cardiac and hepatic function (leukocyte count ⩾3.0 × 10^9^ l^−1^ or absolute neutrophil count ⩾2 × 10^9^ l^−1^; platelet count ⩾100 × 10^9^ l^−1^; haemoglobin ⩾8 g dl^−1^; total serum bilirubin ⩽1.25 × upper limit of normal (ULN) in the absence of liver metastasis or ⩽3.0 × ULN in the presence of liver metastasis; transaminase (ALT, AST) level ⩽3 × ULN in the absence of liver metastasis or ⩽5 × ULN in the presence of liver metastasis; and alkaline phosphatase level ⩽2.5 × ULN). Creatinine clearance was required to exceed 60 ml min^−1^.

Patients were not eligible for study enrolment if they were pregnant, lactating or refused effective contraception, if they had bone metastasis only, known brain metastases or a secondary malignancy, history of another primary malignant disease other than *in situ* carcinoma of the uterine cervix or adequately treated basal cell skin cancer, active infection or any other concomitant severe clinical condition making implementation of the protocol including prehydration difficult. Administration of other cytotoxic, immune or hormonal agents or radiation therapy was not permitted during the study, with the exception of contraceptives, corticosteroids given as antiemetic treatment, or local palliative radiation. Patients were not eligible if they had received prior gemcitabine, vinorelbine, cisplatin or capecitabine. Finally, patients with a history of DPD-deficiency were ineligible for the trial.

### Patient assessment

Patients were evaluated on a regular basis during treatment. The following assessments were performed before each 3-week cycle: physical examination, complete blood count, serum chemistry (including creatinine clearance) and assessment of toxicities. During the initial phase of treatment, complete blood counts were performed twice weekly to determine the nadir values. If the haematological values had not recovered by the time of scheduled treatment, the complete blood count was repeated every week until recovery of leukocyte count to 3.0 × 10^9^ l^−1^ and platelets to ⩾100 × 10^9^ l^−1^.

Baseline tumour assessment was performed within 2 weeks of the start of treatment using imaging procedures, such as ultrasound, computerised tomography or magnetic resonance imaging. Tumour assessments were repeated after every three cycles of therapy, applying the initially used imaging procedure. World Health Organization and NCN-CTC criteria (3.0) were used for the assessment of tumour response and toxicity grading (Ajani *et al*, 1990).

In addition, time to response (time from the start of therapy to first documentation of objective response), duration of response (time from the first documentation of objective response to first evidence of progressive disease), time to tumour progression (time from the start of therapy to first evidence of progressive disease or last follow-up) and survival (time from the start of therapy to death) were measured (intent to treat).

### Treatment schedule

Patients were randomly assigned to one of the following treatment schedules:

1. Gemcitabine 1000 mg m^−2^ (soluted in 250 ml of 0.9% saline) given as a 30-min infusion on days 1+8 followed by vinorelbine 25 mg m^−2^ (soluted in 100 ml of 0.9% saline) given as a 6–10 min infusion on days 1+8 of a 3-week treatment cycle.
2. Gemcitabine 1000 mg m^−2^ (soluted in 250 ml of 0.9% saline) given as a 30-min infusion on days 1+8 plus cisplatin 30 mg m^−2^ (soluted in 500 ml of 0.9% saline) given as a 60-min infusion on days 1+8 of a 3-week treatment cycle. Patients within this treatment arm received pre- and posthydration with 1 l m^−2^ 0.9% saline i.v. over 2 h or orally 2–3 l mineral water over 8 h. Immediately prior to cisplatin 20 mg of furosemide was applied by i.v. injection.
3. Gemcitabine 1000 mg m^−2^ (soluted in 250 ml of 0.9% saline) given as a 30-min infusion on days 1+8 plus capecitabine 1.300 mg m^−2^ (divided in 2 daily doses) given orally on days 1–14 of a 3-week treatment cycle.

All patients received antiemetics (mainly 5-HT_3_ antagonists) according to the local standards. Patients within the cisplatin regimen were allowed to receive corticosteroids also.

Treatment was continued until disease progression or the occurrence of unacceptable toxicity. In case of a partial response or stable disease a maximum of 6 cycles were planned. If patients achieved a complete remission, two additional cycles were allowed (CR+2 cycles; maximum 8 cycles).

### Dose adjustments

Dose adjustments were made on the basis of leukocyte and platelet counts on the day of treatment and clinical assessments of non-haematological toxicities.

A full dose was applied when the leukocyte count was >2.5 × 10^9^ l^−1^, while the platelet count exceeded 100 × 10^9^ l^−1^; the doses of both drugs were reduced by 25% if the leukocyte count was between 2.0 and 2.5 × 10^9^ l^−1^, while the platelet count exceeded 100 × 10^9^ l^−1^; and if the leukocyte count was <2.0 × 10^9^ l^−1^ or the platelet count <100 × 10^9^ l^−1^, the doses of both drugs were omitted. Omitted day-8 doses were not replaced and the next cycle was given timely as scheduled but at reduced doses. If the patient tolerated the dose-modified treatment well, a re-increase of dosage could be attempted in the following cycle. The use of haematopoietic growth factors was allowed in patients with prolonged haematopoietic recovery. If any grade 3 toxicity except nausea/vomiting occurred, drug doses were reduced by 50% while the patient was out of study in case of any grade 4 non-haematological toxicity. A full dose was applied if any grade 0–2 toxicity except nausea/vomiting (grade 3) occurred.

Patients who were randomised to cisplatin were required to have a creatinine clearance, which exceeds 60 ml min^−1^ during treatment, otherwise they were out of study. Creatinine clearance was estimated according to the recommendation of the ‘National Kidney Disease Education Program’ and the ‘Modification of Diet in Renal Disease-study’. The glomerular filtration rate (GFR) was calculated by the MDRD formula: GFR (ml per min per 1.73 m^2^)=186 × C^−1.154^ (mg dl^−1^) × age^−0.203^ (years) × *k*; C=serum creatinine concentration; *k*=correction factor (female 0.742; male 1.0).

### Biometrical analysis

The primary objective of the study was to determine the objective response rate to the study treatment. Secondary end points included time to progression, survival and toxicity.

Simon's optimal two-stage design was used to ensure that the number of patients exposed to this therapy was minimised should the therapy prove ineffective (Simon, 1989). The study was planned to distinguish between a clinically uninteresting response rate of 20% (null hypothesis) and a clinically interesting response rate of 40% (alternative hypothesis). With the type I error being 5% and the type II error 10%, 13 patients were to be enrolled during the first step and an additional 30 patients during the second step. If three or less responses occurred among the first 13 patients or 12 or less responses in the total population of 43 patients, the treatment had to be judged ineffective and enrolment stopped. Assuming a dropout rate of 10%, it was planned to enrol a total of 47 patients on each treatment arm (3 × 47=141 patients).

The determination of the 95% confidential intervals (CIs) resulted by the exact method of Clopper/Pearson.

Time-to-event end points were calculated according to the method of Kaplan and Meier (1959). Patients who received at least one treatment cycle were evaluable for toxicity, and those who had received at least two treatment cycles or those who progressed after the first cycle were evaluable for response.

## Results

### Patient characteristics

**Figure 1:**
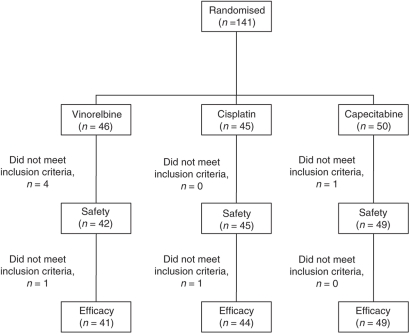
Flow of participants.

One-hundred and forty-one eligible patients were recruited between 2003 and 2006 from 24 German centres. Because of protocol violation in six cases (inclusion criteria) and one written informed consent refusal, a total of 134 patients were evaluated for the primary end point (Figure 1).

Median age was 58 years (GemVin), 60 years (gemcitabine plus cisplatin, GemCis) and 60 years (GemCap), respectively. All patients had previously received prior anthracyclines either in the (neo-)adjuvant or the metastatic setting. Approximately 40% of the patients had received both, an anthracycline- and a taxane-based regimen. Moreover, a majority of the patients presented with visceral metastases (>80%) and ∼75% had more than one metastatic site. About a third of the patients received the study medication as first-line regimen for metastatic disease.

Detailed demography and baseline characteristics are shown in Table 1.

### Treatment delivery

In total, 200 cycles of GemVin, 190 cycles of GemCis and 207 cycles of GemCap were applied. Patients received a median number of 6 cycles (GemVin), 4.5 cycles (GemCis) and 5 cycles (GemCap). Dose reductions and delays were not significantly different among the treatment arms. An increased rate of day-8 omissions contributed to a significantly lower relative dose intensity within the cisplatin combination (81.3 %) compared with 94% within the capecitabine regimen (relative dose intensity, significance test *χ*^2^ for all arms, *P*=0.003; vinorelbine *vs* cisplatin, *P*=0.45; vinorelbin *vs* capecitabine, *P*=0.14; cisplatin *vs* capecitabine, *P*=0.0003).

Detailed information regarding medication is given in Table 2.

### Efficacy – response and survival

With a response rate of 39.0%, 95% CI: 24.2–55.5 (GemVin); 47.7%, 95% CI: 32.5–63.3 (GemCis); and 34.7%, 95% CI: 21.7–49.6 (GemCap), there was no striking difference regarding the primary objective among the three study arms. Overall, the disease control rate (objective response plus stable disease) was 63.4% (GemVin), 56.8% (GemCis) and 59.2% (GemCap), respectively (Table 3).

A detailed analysis of response with regard to triple negative patients was undertaken. The corresponding response rates were 11.1%, 95% CI: 0.3–48.3 (GemVin); 58.3%, 95% CI: 27.7–84.8 (GemCis); and 53.3%, 95% CI: 27.0–78.7 (GemCap).

The median follow-up interval for the whole study population was 11.1 months (95% CI: 7.6–14.6 months). Median duration of response was 6.9 months, 95% CI: 5.1–8.1 (GemVin); 6.9 months, 95% CI: 5.5–8.8 (GemCis); and 8.3 months, 95% CI: 7.1–10.6 (GemCap). The median progression-free survival was 5.7 months, 95% CI: 3.9–8.2 (GemVin); 6.9 months, 95% CI: 5.8–8.8 (GemCis); and 8.3 months, 95% CI: 4.3–9.6 (GemCap), and the median overall survival was estimated with 17.5 months, 95% CI: 12.2–30.0 (GemVin); 13.0 months, 95% CI: 11.0–19.2 (GemCis); and 19.4 months, 95% CI: 16.6–22.0 (GemCap).

**Figure 2:**
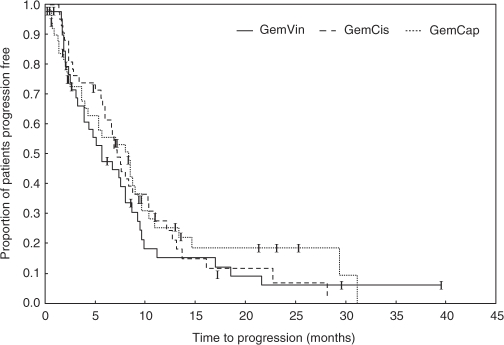
Progression-free survival.

**Figure 3:**
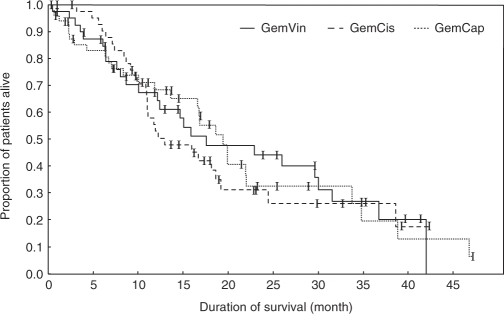
Overall survival.

Progression-free and overall survival curves are shown in Figures 2 and 3.

### Toxicity

The predominant haematological toxicity was grade 3–4 neutropenia, which occurred in 16.6% of the patients within the GemVin arm. This was significantly higher compared with the rate of neutropenia within the GemCap arm (0% *P*=0.004). Moreover, only 4.4% of the patients who received the cisplatin combination experienced grade 3–4 neutropenia. Febrile neutropenia was observed in none of the included patients. The rate of grade 3 and 4 anaemia and thrombopenia was low and comparable within the treatment arms.

Non-haematological toxicity was considered mild to moderate. Grade 3 and 4 non-haematological toxicity was observed in few patients including hand–foot syndrome (2%) and dermatological side effects (4.1%), predominantly in those patients who received capecitabine. Any other non-haematological toxicity was comparable among the study population.

Detailed information of haematological and non-haematological toxicity is given in Table 4.

## Discussion

With the increasing use of anthracycline- and taxane-based regimens in the neoadjuvant and adjuvant setting and their established application in the treatment of the advanced and metastatic stages of breast cancer, there is a clear need for non-cross-resistant further-line regimens.

While there is no established standard of chemotherapy for anthracycline- and taxane-pretreated patients, capecitabine has become a widely accepted agent in this setting. Response rates in the range of 26–52% and time to progression of 3.6–8.9 months were reported in numerous phase II and III trials (Oshaughnessy *et al*, 2001; Ahn *et al*, 2004; Batista *et al*, 2004; Lee *et al*, 2004; Wist *et al*, 2004). The combination of GemCap was investigated in some small phase II studies. Patients who had received that combination as first-line regimen for MBC experienced a high response rate of 61%, whereas the response rate was 41–48.7% for those who had already received anthracyclines and/or taxanes for metastatic disease (Andres *et al*, 2005; Ciruelos *et al*, 2009; Malmstrom *et al*, 2010). With regard to pretreatment, the response rate of 34.7% (95% CI: 21.7–49.6) within the present trial seems to be comparable with these data. The good tolerability of this regimen was documented by the absence of severe neutropenia (grade 3–4 0%) and the low rate of severe hand–foot syndrome (2.0%). Discrepancies regarding the toxicity profile are partly explained by differences within the schedules, as previous investigators reported severe neutropenia in up to 60% of the patients and a consistent rate of hand–foot syndrome of about 15% (Andres *et al*, 2005; Ciruelos *et al*, 2009; Malmstrom *et al*, 2010).

The preclinical rationale for a combination of gemcitabine with a platinum analogue is supported by the synergistic interaction of both agents (Peters *et al*, 1995; van Moorsel *et al*, 1997; Achanta *et al*, 2001). Several clinical studies performed with various schedules have demonstrated that the combination of gemcitabine and platin is highly active not only in first-line treatment, but also in patients previously exposed to anthracyclines and/or taxanes (Nagourney *et al*, 2004, 2008; Nasr *et al*, 2004; Silva *et al*, 2004; Alauddin and Shaharyar, 2005; Burch *et al*, 2005; Stemmler *et al*, 2005; Chitapanarux *et al*, 2006; Demiray *et al*, 2006; Fuentes *et al*, 2006; Heinemann *et al*, 2006; Yardley *et al*, 2006; Laessig *et al*, 2007; Moura *et al*, 2007; Seo *et al*, 2007; Kim *et al*, 2008; Loesch *et al*, 2008; Chew *et al*, 2009; Somali *et al*, 2009). The remission rate of the combination in the present trial was 47.7% (95% CI: 32.5–63.3), which compared favourably to reported remission rates of 21.5–69.2% in the first- and second-line setting published by other investigators (Nagourney *et al*, 2004, 2008; Nasr *et al*, 2004; Silva *et al*, 2004; Alauddin and Shaharyar, 2005; Burch *et al*, 2005; Stemmler *et al*, 2005; Chitapanarux *et al*, 2006; Demiray *et al*, 2006; Fuentes *et al*, 2006; Heinemann *et al*, 2006; Yardley *et al*, 2006; Laessig *et al*, 2007; Moura *et al*, 2007; Seo *et al*, 2007; Kim *et al*, 2008; Loesch *et al*, 2008; Chew *et al*, 2009; Somali *et al*, 2009). As reported by Koshy *et al* (2010), the schedule has proven sustained efficacy with a response rate of 58.3% (95% CI: 27.7–84.8) even in those with triple-negative breast cancer (TNBC). This finding seems to be reaffirmed in the present trial with a response rate of 58.3% (95% CI: 27.7–84.8) in the subgroup of TNBC patients. Regarding treatment-associated toxicity, GemCis must be considered as well tolerable. Compared with previously reported studies, the rate of severe (grade 3 and 4) neutropenia and thrombocytopenia was rather low in our study with 4.4 and 6.7%, respectively.

Finally, the study arm of GemVin within this study yielded a response rate of 39.0% (95% CI: 24.2–55.5), which is also in a range previously published in pretreated patients with MBC (22–55.5% Haider *et al*, 1999; Nicolaides *et al*, 2000; Valenza *et al*, 2000; Stathopoulos *et al*, 2002; Park *et al*, 2009; Shehata *et al*, 2010). Comparably to those studies, the main side effect was grade 3 and 4 neutropenia, but the incidence was considerably lower in our trial (present trial: 16.7, 0% febrile neutropenia *vs* literature: 42–48%, up to 11% febrile neutropenia). Non-haematological toxicity was generally mild, but included grade 3 and 4 nausea and vomiting in few patients with 4.8 and 2.4%, respectively.

In summary, there was no striking difference with regard to any efficacy parameter in terms of response rate, progression-free or overall survival. These results compared favourably with those published by other investigators (Nicolaides *et al*, 2000; Mohran, 2004; Alauddin and Shaharyar, 2005; Andres *et al*, 2005; Burch *et al*, 2005; Stemmler *et al*, 2005; Demiray *et al*, 2006; Fuentes *et al*, 2006; Heinemann *et al*, 2006; Moura *et al*, 2007; Seo *et al*, 2007; Kim *et al*, 2008; Chew *et al*, 2009; Ciruelos *et al*, 2009; Park *et al*, 2009; Somali *et al*, 2009; Koshy *et al*, 2010; Shehata *et al*, 2010). It is important to point out that efficacy is maintained particularly in patients with TNBC (Koshy *et al*, 2010). This applies in particular for the study arms containing cisplatin and capecitabine.

Besides a negligible decrease in dose intensity of the GemCis arm and a slightly increased rate of grade 3 and 4 neutropenia, the toxicity profile among the three study arms seems comparable. None of the recruited patients developed febrile neutropenia, which emphasises the tolerability of the schedules. Comparing all the toxicities that occurred within the present trial with previously published trials that investigated one of the combinations for MBC, there are some strong distinctions regarding the toxicity profile and in particular the rate of severe neutropenia. These discrepancies are partly explained by differences within the doses that were applied in these trials. Therefore, drawing a final conclusion outside a comparative trial is virtually impossible.

In conclusion, this randomised phase II trial has revealed comparable results for all three gemcitabine-based regimens regarding efficacy and toxicity. Gemcitabine-based chemotherapy appears to be a notable treatment option for pretreated patients with MBC.

## Figures and Tables

**Table 1:** Patients demography and characteristics

	**Gemcitabine and Vinorelbine**		**Gemcitabine and Cisplatin**		**Gemcitabine and Capecitabine**	
Randomised patients (*n*)	46		45		50	
Median age (years) (range)	58	(38–77)	60	(36–74)	60	(34–78)
Median KPS (%) (range)	100	(70–100)	95	(70–100)	95	(70–100)
						
	***N***	**%**	***N***	**%**	***N***	**%**
*Tumour metastatic sites*						
Visceral	41	89.1	37	82.2	45	90.0
Non-visceral only	5	10.9	8	17.8	5	10.0
						
*Number of metastatic sites*						
1	12	26.1	13	28.9	12	24.0
2	19	41.3	24	53.3	24	48.0
⩾3	14	30.4	8	17.8	13	26.0
Unknown	1	2.2			1	2.0
						
*Hormone receptor status*						
Positive	28	60.9	26	57.8	25	50.0
Negative	16	34.8	18	40.0	18	36.0
Unknown	2	4.4	1	2.2	7	14.0
						
*HER2 status*						
Positive (IHC3+, FISH+)	4	8.7	7	15.6	5	10.0
Negative	32	69.6	30	66.7	35	70.0
Unknown	10	21.7	8	17.8	10	20.0
Menopausal status						
Premenopausal	10	21.7	8	17.8	4	8.0
Postmenopausal	19^a^	41.3	23	51.1	34^a^	68.0
Unknown	17	37.0	14	31.1	12	24.0
						
*Prior hormonal treatment*						
Prior hormonal treatment	26	56.5	27	60.0	28	56.0
Unknown	2	4.45	—	—	1	2.0
						
*Prior chemotherapy (CTX)*						
Prior anthracyclines	24	52.2	26	57.8	31	62.0
Prior anthracyclines+taxanes	20	43.5	19	42.2	19	38.0
Prior taxanes	1	2.2	—	—	—	—
Unknown	1	2.2	—	—	—	—
Prior (neo)adjuvant CTX	33	71.7	32	71.1	39	78.0
						
* Line of CTX for MBC*						
1st line	18	39.1	15	33.3	18	36.0
2nd line	15	32.6	13	28.9	17	34.0
⩾3rd line	11	23.9	8	17.8	10	20.0
Unknown	2^b^	4.4	9^b^	20.0	5	10.0

Abbreviations: FISH=fluorescence *in situ* hybridisation; HER2=human epidermal growth factor receptor 2; IHC3+=immunohistochemistry (DAKO 3+); KPS=Karnofsky performance scale; MBC=metastatic breast cancer.

a Statistical significant (*P*=0.01).

b Statistical significant (*P*=0.03).

**Table 2:** Medication, dose adjustments and delays

	**Gemcitabine and Vinorelbine**		**Gemcitabine and Cisplatin**		**Gemcitabine and Capecitabine**	
*Number of cycles applied*						
Total	200		190		207	
Median/patient completed (range)	6.0	(1–8)	4.5	(1–8)	5.0	(1–8)
						
	***N***	**%**	***N***	**%**	***N***	**%**
*Doses reduced, delayed or both*						
Day 1	52	26.0	57	30.0	56	27.1
Day 8	56	28.0	58	30.5	61	29.5
						
*Dose omitted*						
Day 1			1	0.5		
Day 8	17	8.5	27	14.2	9	4.4
						
*Dose intensity gemcitabine*						
Planned dose (mg m^−2^ per week)	666.7		666.7		666.7	
Actual median dose (mg m^−2^ per week) (range)	600.0	(307–684)	541.6	(309–679)	618.7	(274–698)
Relative dose intensity (actual/planned × 100)	90.0	(46–103)	81.2	(46–102)	92.8	(41–105)
						
*Dose intensity second substance*						
Planned dose (mg m^−2^ per week)	16.7		20.0		6066.7	
Actual median dose (mg m^−2^ per week) (range)	15.0	(7–17)	16.3	(9–20)	5702.0	(2885–7778)
Relative dose intensity (actual/planned × 100)	90.0	(42–104)	81.3^a^	(46–101)	94.0^a^	(48–128)

a Statistical significant (*P*=0.0003) by Fishers exact test.

**Table 3:** Efficacy – response rates

	**Gemcitabine and Vinorelbine**			**Gemcitabine and Cisplatin**			**Gemcitabine and Capecitabine**		
	***N***	**%**	**95% CI**	***N***	**%**	**95% CI**	***N***	**%**	**95% CI**
CR	3	7.3	1.5–19.9	4	9.1	2.5–21.7	4	8.2	2.3–19.6
PR	13	31.7	18.1–48.1	17	38.6	24.4–54.5	13	26.5	15.0–41.1
ORR	16	39.0	24.2–55.5	21	47.7	32.5–63.3	17	34.7	21.7–49.6
SD	10	24.4	12.4–40.3	4	9.1	2.5–21.7	12	24.5	13.3–38.9
PD	11	26.8	14.2–42.9	11	25.0	13.2–40.3	12	24.5	13.3–38.9
NE	4	9.8	2.7–23.1	8	18.2	8.2–32.7	8	16.3	7.3–29.7
All	41	100.0		44	100.0		49	100.0	

Abbreviations: CI=confidence interval; CR=complete remission; NE=not evaluable by Fishers exact test; ORR=overall response rate; PD=progressive disease; PR=partial response; SD=stable disease.

**Table 4:** Adverse reactions, per-patient-analysis, toxicity grade ⩾3

	**Gem/Vin toxicity grade ⩾3**		**Gem/Cis toxicity grade ⩾3**		**Gem/Cap toxicity grade ⩾3**		**Gem/Vin *vs* Gem/Cis**	**Gem/Vino *vs* Gem/Cap**	**Gem/Cis *vs* Gem/Cap**
	***N***	***%***	***N***	***%***	***N***	***%***	***P*-value**	***P*-value**	***P*-value**
*Haematologic*									
Neutropenia	7	16.7	2	4.4			0.07	0.004	0.49
Febrile neutropenia									
Anaemia	1	2.4	4	8.9	1	2.0	0.36	1	0.19
Thrombopenia	2	4.8	3	6.7	2	4.1	1	1	0.67
									
*Non-haematological*									
Alopaecia									
Fatigue									
Nausea	2	4.8	2	4.4	2	4.1	1	1	1
Vomiting	1	2.4	3	6.7	3	6.1	0.62	0.62	1
Mucositis									
Constipation									
Diarrhoea			1	2.2			1	1	0.47
Infection									
Myalgia									
Sensory neuropathy									
Mot. neuropathy									
Bone pain	1	2.4					0.48	0.46	1
Dyspnoea	1	2.4	5	11.1	2	4.1	0.20	1	0.25
Abdominal pain									
Oedema					1	2.0	1	1	1
Rash									
Hand–foot syndr.					1	2.0	1	1	1
Dermatology					2	4.1	1	0.5	0.5
Creatinine									
ALT (GPT)									
AST (GOT)					3	6.1	1	0.25	0.24
AP	1	2.4			1	2.0	0.48	1	1
Bilirubin					1	2.0	1	0.48	1

Abbreviations: ALT=alanine transaminase; AP=alkaline phosphatase; AST=aspartate transaminase; GOT=glutamate oxalacitate transaminase; GPT=glutamate pyruvate transaminase; MOT=motoric. *P*-values are calculated by Fishers exact test.
